# Review of Current COVID-19 Diagnostics and Opportunities for Further Development

**DOI:** 10.3389/fmed.2021.615099

**Published:** 2021-05-07

**Authors:** Yan Mardian, Herman Kosasih, Muhammad Karyana, Aaron Neal, Chuen-Yen Lau

**Affiliations:** ^1^Indonesia Research Partnership on Infectious Disease (INA-RESPOND), Jakarta, Indonesia; ^2^National Institute of Health Research and Development, Ministry of Health, Republic of Indonesia, Jakarta, Indonesia; ^3^National Institute of Allergy and Infectious Diseases, National Institutes of Health, Bethesda, MD, United States; ^4^National Cancer Institute, National Institutes of Health, Bethesda, MD, United States

**Keywords:** COVID-19, diagnostics, clinical, *in-vitro* assay, molecular test, serologic test, antigen test

## Abstract

Diagnostic testing plays a critical role in addressing the coronavirus disease 2019 (COVID-19) pandemic, caused by Severe Acute Respiratory Syndrome Coronavirus 2 (SARS-CoV-2). Rapid and accurate diagnostic tests are imperative for identifying and managing infected individuals, contact tracing, epidemiologic characterization, and public health decision making. Laboratory testing may be performed based on symptomatic presentation or for screening of asymptomatic people. Confirmation of SARS-CoV-2 infection is typically by nucleic acid amplification tests (NAAT), which requires specialized equipment and training and may be particularly challenging in resource-limited settings. NAAT may give false-negative results due to timing of sample collection relative to infection, improper sampling of respiratory specimens, inadequate preservation of samples, and technical limitations; false-positives may occur due to technical errors, particularly contamination during the manual real-time polymerase chain reaction (RT-PCR) process. Thus, clinical presentation, contact history and contemporary phyloepidemiology must be considered when interpreting results. Several sample-to-answer platforms, including high-throughput systems and Point of Care (PoC) assays, have been developed to increase testing capacity and decrease technical errors. Alternatives to RT-PCR assay, such as other RNA detection methods and antigen tests may be appropriate for certain situations, such as resource-limited settings. While sequencing is important to monitor on-going evolution of the SARS-CoV-2 genome, antibody assays are useful for epidemiologic purposes. The ever-expanding assortment of tests, with varying clinical utility, performance requirements, and limitations, merits comparative evaluation. We herein provide a comprehensive review of currently available COVID-19 diagnostics, exploring their pros and cons as well as appropriate indications. Strategies to further optimize safety, speed, and ease of SARS-CoV-2 testing without compromising accuracy are suggested. Access to scalable diagnostic tools and continued technologic advances, including machine learning and smartphone integration, will facilitate control of the current pandemic as well as preparedness for the next one.

## Introduction

Coronavirus disease 2019 (COVID-19), caused by Severe Acute Respiratory Syndrome Coronavirus 2 (SARS-CoV-2) (1), has dominated the attention of clinicians, researchers, policymakers and communities worldwide. COVID-19 represents the third major spill-over of a coronavirus from animals to humans during the last two decades (2), with greater global impact than the previous coronavirus outbreaks in 2003 (SARS-CoV) and 2012–2015 and 2020 (Middle East Respiratory Syndrome Coronavirus/MERS-CoV). Transmission of SARS-CoV-2 may have been enhanced by spread from asymptomatic and mildly symptomatic individuals, as opposed to SARS-CoV and MERS where patients tended to be sicker and less mobile, thus resulting in a higher basic reproduction number (R_0_) for SARS-CoV-2 (3–6). First reported in China, SARS-CoV-2 spread globally within months, with the Americas, South Asia, and Europe being most severely affected to-date. As of end-March 2021, there were more than 125 million confirmed cases and over 2.7 million deaths, reflecting a global case fatality rate of 2.19% (7), compared to 8,096 total cases and 774 confirmed deaths for SARS, and 2,521 total cases with 866 confirmed deaths for MERS (3). As of Feb 21, 2021, U.S. deaths from COVID-19 had surpassed the death toll of its citizens from World War II, the Korean War, and the Vietnam War combined (8).

In response to the rapidly evolving COVID-19 pandemic, a variety of testing approaches have been employed based on local testing capacities, public health resources, and epidemiology. Large-scale testing, in combination with contact tracing and broad public health control measures, has proven effective in containing SARS-CoV-2 in South Korea and Taiwan (9–11). However, resource limitations in some regions and poor external validation of newly developed diagnostic assays create challenges for successful containment and mitigation (12). The ever-expanding list of diagnostics under the U.S. Food and Drug Administration (FDA)'s emergency use authorization (EUA) also contributes to the confusion around test selection, as performance characteristics, infrastructure requirements, and global availability vary. We herein review available COVID-19 diagnostic approaches, with a focus on their underlying principles and indications, and explore ways in which application of these diagnostics might be improved.

Diagnostic approaches to COVID-19 can be divided into two broad categories: Clinical diagnostics and *in vitro* diagnostics (12–14). Clinical diagnostics include symptoms, laboratory markers not specific to SARS-CoV-2, and imaging, all of which may raise suspicion of COVID-19 but do not provide definitive evidence (13). *In vitro* diagnostics consist of nucleic acid amplification tests (NAATs) and serologic antibody and antigen-based assays, which are specific to SARS-CoV-2 and are broadly applicable in the different settings of clinical care, public health, or epidemiologic investigations (15). *In vitro* diagnostic assays are recommended by the U.S. CDC and U.S. NIH for people who have symptoms of COVID-19, close contact (within 6 feet) with a confirmed case, have participated in higher risk activities where social distancing is not possible, or who have been referred for testing by a healthcare provider or health department (16). Individuals without symptoms or exposure risks are not currently prioritized for testing but may be screened for other reasons such as public health monitoring, active surveillance, or compliance with state and local plans (15, 16).

## Clinical Diagnostics

Clinical diagnostics for COVID-19 include the initial assessment of possible COVID-19 related symptoms and exposure history. These should be considered in the context of the SARS-CoV-2 incubation period, which is estimated to be up to 14 days from exposure, with a median of 4–5 days (17–19). Eleven common symptoms of COVID-19 are noted by the U.S. CDC: fever or chills, cough, dyspnea, fatigue, muscle pain, headache, new loss of taste or smell, sore throat, congestion or runny nose, nausea or vomiting, and diarrhea (20). Hospital admission data suggests that fever and cough are the most frequent manifestations (17, 21, 22), and the WHO interim guidance updated on August 7th, 2020, emphasized recent anosmia or ageusia as specific for COVID-19 (23, 24). These observations may be related to high expression of the SARS-CoV-2 host receptor angiotensin-converting enzyme 2 (ACE2) in the nasopharynx (24, 25) or spike protein mutations (D614G) that augment local replication (26).

More recent data also suggest that conjunctivitis, dermatologic findings (maculopapular and vesicular lesions), and multisystem inflammatory syndrome in children (MIS-C), which clinically resembles Kawasaki disease, are associated with infection (27–29). Acute strokes and myocardial infarctions have also been reported, indicating multi-organ involvement that is being further evaluated in several studies (30, 31). A clinical prediction model based on eight factors (cough, fever, contact with a confirmed case, gender, age 60+, headache, sore throat, and shortness of breath) independent of RT-PCR has been developed by the Israeli Ministry of Health, with 87.30% sensitivity and 71.98% specificity (32). Validation of the model in a larger cohort is needed to improve generalizability and evaluate the need for inclusion unique COVID-19 symptoms such as anosmia and ageusia.

Radiography may also support clinical suspicion of COVID-19, and chest CT scanning has been used as a complementary approach for early diagnosis and evaluation of disease progression. CT scan findings are variable and can include multiple bilateral ground-glass opacities in the peripheral lower lung zones (33), which are also seen in patients with SARS-CoV and MERS-CoV infections (34, 35). In 1,014 patients in Wuhan, China, who underwent both RT-PCR testing and chest CT scanning, a “positive” chest CT scan for COVID-19 (per consensus of two radiologists) had a sensitivity of 97% when using RT-PCR as the reference, though specificity was only 25% (36). False-positive CT scan interpretation is not unexpected since findings overlap with other causes of pneumonia (37). Additional studies highlight chest radiograph findings (hazy opacities, consolidation, or horizontal linear opacities) (23, 38) and point-of-care ultrasound pathology (thickened pleural lines, fused B lines, comet-tail artifact or consolidation patterns with or without air bronchograms) (23, 39) as common features of COVID-19. Nonetheless, COVID-19 patients may not show radiographic abnormalities (38, 40).

Laboratory biomarkers, like radiography, are non-specific for COVID-19 but may also contribute to clinical suspicion of the disease. Reliance upon widely available markers was especially common early in the pandemic, when specific testing capacity was extremely limited (17, 41). Common laboratory findings amongst COVID-19 patients include leukopenia, lymphopenia, elevated aminotransaminase levels, elevated lactate dehydrogenase (LDH) levels, and elevated inflammatory markers (e.g., ferritin, C-reactive protein, and erythrocyte sedimentation rate) (22, 42). Correlation between laboratory findings, disease severity, comorbidities and complications continue to be investigated (43). High D-dimer levels, severe lymphopenia, increased neutrophil to lymphocyte ratio, marked thrombocytopenia, hypoalbuminemia, elevated IL-6, procalcitonin, cardiac troponin I, and serum amyloid A are associated with critical illness or mortality in COVID-19 (44–48). However, these non-specific biomarkers may also be elevated in other infectious diseases such as dengue fever, typhoid fever, or influenza (49, 50).

Artificial intelligence (AI) has also shown promise for automated detection of COVID-19 via pattern recognition algorithms and may potentially reduce emergency department workloads (51). Radiology has been an early adopter of AI for disease detection. In one multisite study, AI deep learning on CT images was able to distinguish COVID-19 from other causes of pneumonia (AUC = 0.87 and 0.88) (52). AI systems based on chest X-ray images showed a sensitivity of 94.8% (53) and accuracy of 96% (54) for prediction of COVID-19 pneumonia. Radiologic data alone may not be suitable for ruling out COVID-19, especially during early disease. Machine learning that integrates chest CT findings with clinical symptoms, exposure history and laboratory testing shows promise for rapid COVID-19 diagnosis (55). An AI model achieved an AUC of 0.92, with sensitivity of 84.3% and specificity of 82.8% (55). Machine learning integration with a smartphone-based application has been proposed for COVID-19 self-testing using breathing or cough sounds; it recognizes acoustic patterns to diagnose COVID-19 early (56, 57). Real-world data should be collected to validate this approach.

AI deep learning has also been used to analyze species specificity of volatile organic compounds (VOCs) by breath-biochemistry, potentially providing a species level biological fingerprint for the pathogen (58). Sensitivity of breath-analyzer tests for COVID-19 ranges from 82.4 to 100% and specificity 54–90% (59–61). False-positives are influenced by diet, humidity, and background contamination (59, 61). Despite its relatively low specificity and need for validation, AI-based breath tests could become a quick, low-cost, and non-invasive triage tool for excluding COVID-19 in the future (60).

## *In*-*vitro* Diagnostics: Molecular Testing

SARS-CoV-2 infection is confirmed by detection of SARS-CoV-2 RNA using NAAT (62). For detecting RNA viruses like SARS-CoV-2, Reverse Transcription quantitative PCR (RT-PCR) is recommended as the most sensitive NAAT method (63, 64). Conventional NAAT begins with RNA extraction from respiratory specimens, followed by RT-PCR, in which the purified total RNA (viral RNA and the host RNA) is reverse transcribed into complementary DNA (cDNA) first by reverse transcriptase, followed by cDNA aliquots undergoing qPCR to exponentially amplify the target gene of interest (15, 63, 65). This two-step assay usually takes 3.5–4.0 h and requires three reagent kits: one for the RNA extraction, one for cDNA synthesis, and another for the amplification and detection of the target nucleic acid, as well as specialized lab equipment (15). Throughout the pandemic, labs have faced global shortages of diagnostic reagents, particularly for RNA extraction, and personal protective equipment (PPE) for personnel at risk of exposure in the lab (66). Simplification of NAAT by removing the RNA extraction step is being explored (67). Reports suggest that skipping RNA extraction by simple direct heating of specimens for 5 min at 98°C results in sensitivity and specificity comparable with standard methods (68). Others have successfully processed fresh undiluted samples at 99°C for 5 min (69) or 70°C incubation for 10 min (66) without an RNA extraction step. However, optimization of analytical sensitivity across specimen types remains one of the greatest challenges.

Systems that automate nucleic acid extraction, purification, amplification and detection are available. These provide rapid, high-throughput results with minimal hands-on time (HoT) and less contamination (70–72). The Cobas® SARS-CoV-2 6,800 and 8,800 systems (Roche Molecular Diagnostics, Pleasanton, CA, USA) have sample throughputs ranging from 96 results in 3 h to 384 results (6,800 system) or 1,056 results (8,800 system) in 8 h (70, 73). Overall agreement with standard RT-PCR is up to 99.6% (74). Abbott Molecular (Des Plaines, IL, USA) has also developed a high-throughput, fully automated assay that runs on the m2000 system. This system processes up to 96 samples simultaneously and reports 470 test results in ~24 h, with high sensitivity (93%) and specificity (100%) for detecting SARS-CoV-2 in clinical samples compared to the SARS-CoV-2 RT-PCR assay developed by the U.S. CDC (75).

Three other automated sample-to-answer assay platforms developed during the pandemic are the Hologic Panther Fusion SARS-CoV-2 assay (Hologic, Inc., San Diego, CA), the Hologic Aptima SARS-CoV-2 assay (Hologic, Inc., San Diego, CA), and the BioFire Defense COVID-19 test (BioFire Defense, Salt Lake City, UT) with throughputs of 335, 275, and 72 samples in 8 h, respectively (72, 76). The Fusion and BioFire automate all aspects of nucleic acid testing including sample preparation, nucleic acid extraction and PCR amplification using nested multiplex PCR, while the Aptima assay uses target capture and Transcription Mediated Amplification (TMA) for the isolation and amplification of SARS-CoV-2 RNA (77–79). Despite slight differences in SARS-CoV-2 target regions and NAAT method, they showed comparable clinical performance for detection of SARS-CoV-2 in NP swabs. Compared to the consensus result (positive for ≥2 of 3 NAATs), the Fusion and BioFire assays had a positive percent agreement (PPA) of 98.7%, followed by the Aptima assay at 94.7%. All 3 assays demonstrated 100% negative percent agreement (NPA), suggesting high specificity (76).

Laboratories facing reagent shortages have sometimes implemented multiple platforms to augment specimen processing capacity. As different platforms employ different techniques and expertise, simultaneous use of diverse platforms could result in inadvertent errors. Additional personnel may also be required, which can create undesirable crowding. And inefficiencies in processing can occur as technicians multitask between analyzers, resulting in increased turnaround time (TAT) (80). Unfortunately, availability of automated RT-PCR for high-throughput platforms remains critically limited, especially for low- and middle-income countries (81).

### Specimens for Molecular Testing

SARS-CoV-2 NAAT is most commonly performed on upper respiratory samples. The U.S. CDC recommends that swabs be obtained from the nasopharynx (NP), oropharynx (OP), nasal mid-turbinate, or anterior nares. Wash or aspirate from the nares or NP is also appropriate (82). Samples should be collected by health care providers using a flocked swab with an aluminum or plastic shaft to enhance collection and release of cellular material. Swabs containing calcium alginate or wooden shafts are known to contain PCR inhibitory substances that can lead to false-negative results and should be avoided (63, 83). Swab specimens should be placed into universal transport medium (UTM) immediately after collection to preserve viral RNA (84). Comprehensive data is unavailable for comparing performance of different upper respiratory specimens, though some studies suggest that NP swabs are more sensitive and accurate than OP swabs (85, 86). Compared with standard NP specimens, less invasive nasal swabs (87) and nasal-mid turbinate specimens (88) may cause less discomfort and greater compliance, though at the expense of diagnostic accuracy. Upper respiratory samples have been the leading candidates for home testing thus far.

Due to a global swab shortage, discomfort associated with NP collection, need for trained healthcare personnel, and risk of aerosol droplet production, there is great interest in alternatives to NP specimens. Saliva is a leading candidate, as SARS-CoV-2 RNA is reliably detected within the first week of symptom onset (89). Saliva testing demonstrates similar sensitivity to NP specimens for the detection of SARS-CoV-2 during hospitalization (90). Salivary viral load also correlates with other biological markers such as LDH and may provide information about the clinical evolution of COVID-19 (91). In response to resource shortages and long testing delays, specimen pooling has been used as a large-scale testing strategy (92). Pooling is most efficient when SARS-CoV-2 infection incidence is low, as demonstrated in a study where testing capability was increased at least 69% when one positive swab was mixed with four negative SARS-CoV-2 specimens (93). Use of alternative specimens and modification of testing approaches to increase throughput should be further evaluated to ensure that performance compared to gold-standard RT-PCR is not compromised.

Lower respiratory tract specimens (tracheal aspirates, bronchoalveolar lavage (BAL), fibrobronchoscopic brush biopsy, or sputum) are also valuable for diagnostic testing, as they demonstrate higher positivity rates than upper respiratory specimens, especially later in disease course (94). A non-invasive Exhaled Breath Condensate (EBC) technique that samples respiratory droplets from the lower respiratory tract is being explored for COVID-19 molecular testing. However, EBC should only be used as an adjunct, as opposed to replacement, for NP RT-PCR due to inconsistent results thus far (95). Non-respiratory samples such as blood, feces, urine, semen, or cerebrospinal fluid (CSF) have been used, though their interpretation remains controversial (96–101). Infectious virus has been isolated from urine and feces, but the presence of RNA in non-respiratory specimens does not necessarily correlate with COVID-19 severity, local symptoms (e.g., diarrhea or urinary tract symptoms), or mode of transmission (98–101).

Stool has been considered for COVID-19 testing. SARS-CoV-2 RNA was detected in stool in 48.1% of patients during the course of illness but persisted longer than in respiratory samples (102). In a recent systematic review and meta-analysis, the mean duration of SARS-CoV-2 RNA shedding was 17.0 days (95% CI 15.5–18.6; 43 studies, 3,229 individuals) in the upper respiratory tract, 14.6 days (95% CI 9.3–20.0; seven studies, 260 individuals) in lower respiratory tract, and 17.2 days (95% CI 14.4–20.1; 13 studies, 586 individuals) in stool (103). An earlier study highlighted two COVID-19 cases with positive stool before pharyngeal specimens (102), suggesting that stool may be an alternative to respiratory specimens for early virus discovery in individuals unable to provide respiratory samples, such as infants (104). Stool as a source is consistent with the virus being found in wastewater, where it is presumed to survive several days. During the March–April 2020 Paris COVID-19 outbreak, SARS-CoV-2 levels in waste-water tracked the increase of regional COVID-19 cases observed (105). Thus, sewage–waste-water monitoring could be a non-invasive surveillance strategy (63, 106).

SARS-CoV-2 was also found in 15.8% of semen samples from 38 men with COVID-19 (107), and RNA has been detected in CSF despite its absence in NP swabs in a COVID-19 patient with meningitis/encephalitis (108). Lastly, it has been postulated that COVID-19 begins with circulating viremia before progressing to pneumonia (109), but the presence of SARS-CoV-2 RNA in blood remains unclear (99). Detection of SARS-CoV-2 in blood has ranged from 1 to 8%, and its presence may be associated with increased clinical severity (94, 99, 110). Systematic analysis (108 individuals) showed mean duration of SARS-CoV-2 RNA shedding in serum was 16.6 days (95% CI 3.6–29.7), and the maximum shedding duration was 60 days (103). However, one small study was unable to culture virus from 27 RT-PCR-positive serum samples (111). Correlations between specimen types in which SARS-CoV-2 is detected and organ system manifestations should be further explored.

### Technical Aspects of Molecular Testing

Isolation of RNA is the initial step of the RT-PCR assay and critical for the assay's reproducibility and biological relevance (63). Unlike DNA, RNA is highly susceptible to degradation; sample storage, handling, and RNA isolation must follow optimized protocols to minimize degradation at each step (63, 112). After RNA purification, reverse transcription is conducted using different primers, including oligo-dT, random, or gene-specific, depending on the type of RNA, cDNA yield, and specificity (113). Both the SARS-CoV-2 viral RNA and human RNA (host control RNA such as RNase P) are reverse transcribed; the same cDNA can be used for qPCR (63).

One-step and two-step RT-PCR assays are commercially available. In a one-step assay, reverse transcription and PCR amplification are consolidated into one reaction utilizing a single tube and buffer for RT and PCR steps. In a two-step assay, the reactions are done sequentially in separate tubes with independently optimized buffers (65, 114). One-step RT-PCR can provide rapid and reproducible results, is suitable for high-throughput diagnosis, and may reduce risk of cross-contamination and human error by limiting sample management (12). On the other hand, the more time consuming two-step RT-PCR offers superior sensitivity and lower detection limits (115).

RT-PCR should target highly conserved and abundantly expressed genes of SARS-CoV-2 (62). Positive and negative controls are also important for quality assurance (63). Samples spiked with synthetic SARS-CoV-2 RNA or previously validated positive samples may serve as positive controls (63). Internal “house keeping” control (IC) reactions such as human RNase P mRNA should be included to minimize false negatives associated with technical errors (63, 116). Failure to detect the RNase P gene may indicate improper RNA extraction, RNA degradation/loss, insufficient human cellular material, or reagent or equipment malfunction (63).

Different institutions rely on varying numbers of SARS-CoV-2 gene targets and different target regions. Gene targets include structural proteins, which have higher sensitivity for coronavirus detection, and species-specific SARS-CoV-2 accessory genes (104). Use of multiple PCR targets helps to avoid false-negatives associated with mutations in the primer site, especially mismatches at the 3' end (117, 118). The structural spike (S), nucleocapsid (N), non-structural RNA-dependent RNA polymerase (RdRp), and the open reading frame ORF1ab are the most commonly targeted genes (15).

The U.S. FDA and CDC recommend assays detecting viral nucleocapsids N1 and N2 and human RNase P genes as the primary targets and internal control (IC), respectively (119). A cycle threshold (Ct) value of <40 for all target genes is defined as a positive test, while a Ct value <40 for only one of the two nucleocapsid proteins is considered indeterminant and requires confirmation by retesting (15). This approach differs from the WHO assay, which employs the Charité, Berlin, two-step assay algorithm to confirm infection: step one screens for the envelope (E) gene of subgenus Sarbecovirus, and step two screens for the RdRp gene, which is highly specific for SARS-CoV-2 and does not cross-react with other coronaviruses (120). China CDC recommends the use of specific primers and probes in the N gene regions and the ORF1ab, which encodes a replicase polyprotein 1ab required for viral RNA replication and transcription. Infection is considered confirmed when both targets are positive (37). Other countries have adopted different viral targets for PCR detection: the Pasteur Institute of Paris targets two regions within the RdRp gene; the National Institute of Health, Thailand, and the National Institute of Infectious Disease, Japan mainly uses the N gene; and Hong Kong health authorities target ORF1b-nsp14 and the N gene (114, 121).

Recent studies comparing performance of RT-PCR assays using different target regions have shown that N and E gene primer-probe assays are more sensitive than RdRp based assays (116, 122–124). The lower sensitivity of the RdRp based assay may be due to a mismatch in the reverse primer (122). However, these findings could be confounded by use of different PCR systems, relatively small sample size, and lack of phylogenetic analysis (116). To improve diagnostic efficiency and reliability, duplex or multiplex real-time RT-PCR tests have been developed. These allow simultaneous detection of two or more target sequences via specific fluorescent-labeled probes (65). For instance, the FDA emergency use authorized Abbott RealTime SARS-CoV-2 assay is a dual target RT-PCR assay that detects RdRp and N genes; the TaqPath^TM^ COVID-19 Combo Kit by Life Technologies (Thermo Fisher Scientific, Inc.) employs quantitative recognition of ORF1ab, N, and S genes simultaneously (125, 126). However, the CDC and WHO recommend separating internal control reactions as opposed to multiplexing them in the same PCR reaction with SARS-CoV-2 target genes because relatively high levels of human RNase P RNA compared to SARS-CoV-2 viral RNA may reduce sensitivity of SARS-CoV-2 target genes when multiplexed in one reaction (63).

Like all diagnostic tests, false-negative results can occur with RT-PCR. False negatives have been reported to occur in ~30% (range 10–40%) of patients with COVID-19 (15). Contributing factors may include (a) collecting the sample when the viral load is low (e.g., early after exposure and before the peak associated with symptom onset, or late in disease course), (b) sample collection technique resulting in reduced quality or quantity, (c) inadequate preservation of the unstable RNA virus, as specimens may degrade without appropriate transport medium or storage, and (d) technical limitations of the RT-PCR test (3, 15, 127–130). One pooled analysis found the probability of a false-negative result ranged from 100% on day 1 after infection to 21% on day 9 to 66% on day 21 (129). False-negative results might be addressed by adjusting the timing of swab collection and repeat testing in the context of high suspicion (12). Positive stool PCR tests with negative pharyngeal swabs have been reported in patients with predominantly GI symptoms. Thus, anal sampling has been considered when there are concerns that NP testing may be falsely negative (131–133). Interpretation of anal specimens should take into account that prolonged nucleic acid does not necessarily reflect presence of infectious virus. Furthermore, testing should not be eschewed to improve rates of case detection, but must be tailored to public health needs.

Test sensitivity may be impacted by natural mutations in the primer region, which could result in false-negatives (134). Based on sequence analysis of the SARS-CoV-2 genomes submitted to the Global Initiative on Sharing All Influenza Data (GISAID) database, viral mutation was highest in the China-CDC-N primer regions compared to other primer sets (https://www.gisaid.org/) (135). Though this does not necessarily mean that a primer would fail to bind, it reveals variability of the target region. It is unclear whether primers for SARS-CoV-2 should be updated regularly as with influenza. One study reported association between a C-to-U transition at position 26,340 of the SARS-CoV-2 genome and failure of the Cobas SARS-CoV-2 E gene RT-PCR in eight patients (118). Another report showed deletion in S-gene positions 69 and 70 in the Variant of Concern (VOC) 202012/01 or B.1.1.7 causes S-gene target failure (SGTF) in at least one RT-PCR–based diagnostic assay, the ThermoFisher TaqPath COVID-19 assay, and may serve as a means pf identifying infection with this variant (136). These findings highlight the need for ongoing assessment of RT-PCR targets.

Viral RNA detection by RT-PCR does not demonstrate the presence of infectious virus, and patients who have recovered can be persistently PCR-positive but non-infectious, which is confusing for quarantine and control (137). Cell culture is a more accurate indicator of viability and contagiousness but must be performed in Biosafety Level 3 (BSL-3) facilities and is not routine (101, 138). Studies have shown that RT-PCR Ct values correlate strongly with the ability to cultivate virus (139–141). However, Ct value cut-offs differ between studies and depend on the PCR system used. Variation across PCR test runs, low viral copy number, and poor sampling collection may engender differences in absolute Ct values (142–144). Some studies have shown that the probability of culturing virus declines to 8% in samples with Ct > 35 by RT-PCR targeting RdRp gene (141), while other studies have concluded that patients with Ct > 33–34 by LightCycler Multiplex RNA Virus Master kit RT-PCR system targeting E gene are not contagious (140). Others have even provided data showing no virus growth in samples with Ct > 24 of E gene amplification by RT-PCR (139). Duration of illness negatively affects the viability of SARS-CoV-2 in specimens, as isolates have resulted in no growth when collected after day 8 of illness despite ongoing high viral loads by RT-PCR (97, 139). Surrogate methods to identify infectious virus, such as the detection of sub-genomic RNA (sgRNA) are being evaluated (144). Additional large-scale studies will be useful for the optimization of strategies to detect infectious virus, which would be helpful for guiding isolation polices.

### Point of Care Molecular Diagnostic Tests

COVID-19 cases are typically confirmed by centralized RT-PCR testing in certified labs, which requires expertise, specialized equipment, and well-developed specimen management infrastructure. Due to the burden of large-scale testing suddenly placed on most labs, results may take a week or longer to be returned. This has spurred significant interest in reliable PoC molecular tests that produce rapid results (<1 h) (81), as they facilitate timely patient management decisions. At least two cartridge-based PoC assays have been developed to-date and granted an EUA from the U.S. FDA (72, 145, 146).

Xpert® Xpress SARS-CoV-2 test (Cepheid, Sunnyvale, CA), the most popular PoC test thus far, provides qualitative detection of the virus in ~45 min using the GeneXpert benchtop system. This PoC NAAT for upper respiratory specimens requires <1-min HoT for sample preparation and targets the N2 and E genes of SARS-CoV-2. The Xpert® Xpress SARS-CoV-2 test demonstrated 100% agreement with in-house RT-PCR assays, with a lower limit of detection (LOD) of 8.26 copies/mL (147). Just as the GeneXpert Assay for tuberculosis (TB) is used for the detection of both wild-type and rifampicin-resistant TB (148), it is anticipated that Xpert COVID-19 could be further developed to detect mutations of SARS-CoV-2 which might impact prevention and treatment approaches.

The second PoC molecular assay under a U.S. FDA EUA is the ID Now COVID-19 test (Abbott Diagnostics Scarborough, Inc., Scarborough, ME). This automated test qualitatively detects SARS-CoV-2 RNA from upper respiratory specimens. ID Now COVID-19 uses an isothermal nucleic acid amplification test (INAAT) based on Nicking Enzyme-Assisted Reaction (NEAR) technology (149) to amplify the RdRp gene in 5–13 min, with a LOD of 125 genome equivalents/ml according to the manufacturer (150). However, the test is limited to only one sample per run, and it showed a sensitivity of only 80.4% and a specificity of 95.9% in a diagnostic confirmation study (151). The lower PPA occurred more frequently in specimens with low viral load or collected in universal or viral transport media (VTM), which may dilute the sample and decrease sensitivity. Therefore, the manufacturer recommends the use of freshly collected specimens for optimal performance (152, 153). However, a small study reported low PPA of ID Now compared with Xpert® Xpress irrespective of use of dry nasal swabs or swabs in VTM, which raises concerns about the suitability of ID Now as a confirmatory diagnostic (150). Due to its suboptimal sensitivity, several institutions have abandoned Abbott ID NOW for POC COVID-19 testing. The U.S. FDA also recommends confirming all negative Abbott ID NOW SARS-CoV-2 results with a sensitive molecular test (154).

Another cartridge-based PoC that has received the Europe CE mark is CovidNudge (DnaNudge, UK), a fully-automated multiplex RT-PCR system with a sample-to-answer run-time of <90 min. This assay uses dry NP swabs and targets seven SARS-CoV-2 gene regions (RdRp1, RdRp2, E-gene, N-gene, N1, N2, and N3) and a validated positive control host gene (RNase P), which reduces the false-negative testing rate caused by insufficient sampling. The overall sensitivity is 94% (95% CI 86–98), with an overall specificity of 100% (99, 100), and LOD 250 viral copies/swab (155, 156). However, since each unit can process only one cartridge at a time (maximum of 15 tests per machine per day), the assay has relatively low throughput and may require multiple processing units (Nudgebox) in a clinical setting (157). Prospective studies are required to assess the effectiveness of CovidNudge with non-NP/OP specimens and in comparison with other standard tests.

Truenat (Molbio Diagnostics, India) was recently developed by Indian scientists via adaptation of a test used for pulmonary tuberculosis (158). This chip-based portable PCR is intended to facilitate quick and affordable molecular pathogen detection by low infrastructure health facilities in developing countries (63). The Truenat Beta CoV E-gene screening assay and Truenat SARS- CoV-2 RdRp gene-confirmatory assay have demonstrated concordance with the reference standard RT-PCR (159). In a small validation study, this PoC assay exhibited 100% sensitivity and specificity and no cross-reactivity with other respiratory pathogens with LOD 486 copies/mL (160, 161). Although the technology lacks the throughput of the conventional PCR, its affordability, portability, ease of use, and test interpretation make it attractive for screening and confirmation of SARS-CoV-2 in developing countries (63).

BioFire® Respiratory Panel 2.1 (RP2.1) (BioFire Diagnostics, Biomérieux, France) is another widely used testing platform. This PoC test uses a closed disposable system containing the reagents necessary for sample preparation, reverse transcription, polymerase chain reaction (PCR), and detection of nucleic acid from multiple respiratory pathogens based on a single NP specimen. Runs take ~45 min (72). RP2.1 was created by adding primers for the membrane (M) and spike (S) genes of SARS-CoV-2 to the existing FDA-cleared and CE-marked BioFire® Respiratory Panel 2 (RP2); RP2.1 can detect 22 viral and bacterial respiratory pathogens with LOD 500 copies/mL for SARS-CoV-2 (162, 163). A study comparing the BioFire RP2.1 with Roche Cobas, Hologic Fusion, and conventional RT-PCR for detection of SARS-CoV-2 demonstrated 98% PPA and 100% NPA in residual NP swab specimens (163). As RP2.1 detects spike genes, a hotspot for mutation, utility of this PoC test for detection of variants should be routinely assessed.

The cobas® Liat® SARS-CoV-2 and influenza A/B test (Roche Molecular Systems, Inc., Pleasanton, CA) has received emergency use authorization (EUA) for identification and differentiation of SARS-CoV-2, influenza A virus, and influenza B virus. This PoC test is a multiplex RT-PCR and provides results in ~20 min (72). For SARS-CoV-2, the test utilizes two target gene regions (ORF1a/b and N) with LOD 12 copies/mL (164). A multisite U.S. study demonstrated excellent test agreement (100% PPA and 97.4% NPA) between the Liat and high-throughput Cobas® 68/8800 tests (165). The Liat is advantageous in that it simultaneously tests for influenza and SARS-CoV-2, allowing differentiation between multiple respiratory viruses that co-circulate (165). Given influenza's ability to exacerbate SARS-CoV-2 infection, early and rapid detection of SARS-CoV-2 and Influenza co-infection may reduce associated morbidity and mortality (166).

Nanomaterial-based biosensors have been developed as a potential PoC approach. These alternatives to viral RNA extraction and SARS-CoV-2 sequence detection use biosensors functionalized with nucleic acid hybridization (167, 168). The GenMark ePlex SARS-CoV-2 (GenMark Diagnostics, Carlsbad, CA), a PoC test based on the eSensor technology (169, 170), is a “True Sample-to-Answer Solution” that targets the N gene. It uses a combination of electrowetting and GenMark's eSensor technology for extraction, amplification, and detection. The technology relies on competitive DNA hybridization and electrochemical detection (155, 171). While the ePlex SARS-CoV-2 Test only detects 1 viral target, the ePlex Respiratory Pathogen Panel 2 (ePlex RP2 Panel) simultaneously detects 16 respiratory viral targets and two bacterial targets (155, 169, 172). The sample-to-result time for both tests is under 2 h, with SARS-CoV-2 LOD 750 genomic copies/mL for ePlex and 250 genomic copies/mL for ePlex RP2 Panel (155, 169, 172).

Simplexa™ COVID-19 Direct assay (Diasorin Molecular LLC, Cypress, CA) is another PoC test available under U.S. FDA Emergency Use Authorization (173). The system consists of the Simplexa™ COVID-19 Direct assay, the LIAISON® MDX (with LIAISON® MDX Studio Software), the Direct Amplification Disc (DAD), and associated accessories. A 50-μl volume of Simplexa COVID-19 Direct kit reaction mix (MOL4150) is added to the “R” well of the 8-well DAD followed by addition of 50 μl of non-extracted NP specimen to the “SAMPLE” well. The assay runs for ~90 min (155, 173, 174). It targets the ORF1ab and S genes and has a LOD for NP specimen of 500 copies/mL (173). The Simplexa and ePlex assays have similar HoT and TAT, based on processing 8 samples per disc on the DiaSorin LIAISON MDX and 6 cartridges per tower in the GenMark ePlex (174). A study evaluating the analytical and clinical performance of the Simplexa, ePlex, Hologic Fusion, and modified CDC conventional RT-PCR showed comparability (κ ≥ 0.96). PPA was 100% (51/51) for Simplexa, Hologic Fusion and conventional RT-PCR and the ePlex PPA was 96% (49/51) compared to the consensus result (positive for ≥3 of 4 NAATs). An NPA of 100% (53/53) was observed for ePlex and Simplexa; NPA ranged from 98% (52/53) for conventional RT-PCR to 96% (51/53) for Hologic Fusion (174).

While both PoC platforms and automated high-throughput systems (e.g., Hologic Fusion) out-performed conventional RT-PCR in hands-on and manual workflow steps, the high-throughput system is more appropriate for high-volume testing since pipetting of specimen into lysis tubes can be labor-intensive and time-consuming, thus may increase TAT. Both PoC and high-throughput assays are suitable for facilities with low to moderate testing volume and need for rapid results (174). Further studies are needed to determine their performance in comparison with gold standard RT-PCR and clinical utility, especially with regards to emerging variants.

### Other Methods of Viral RNA Detection

Although conventional RT-PCR is currently the gold-standard in SAR-CoV-2 diagnosis, it can be time-consuming, laborious, and require specialized equipment and trained personnel (63). Loop-mediated isothermal amplification (LAMP) combined with reverse transcription (RT-LAMP) has been developed as an alternative (114). RT-LAMP is a highly specific assay that employs DNA polymerase and 4–6 primers that bind distinct target regions of the genome; it allows direct detection of SARS-CoV-2 genes such as ORF1ab, S, E, and/or N gene (175–178). RT-LAMP isothermally (60–65°C) amplifies DNA fragments of interest, thus does not require expensive thermal-cyclers or real-time PCR (179). Detection is based on photometric measurement of turbidity resulting from magnesium pyrophosphate precipitation that occurs as a by-product of amplification. This method enables real-time monitoring of results using colorimetric or fluorescent dyes (43, 180). Since RT-LAMP needs only heating and visual monitoring and has a sample-to-result time of around 1 h, it is an attractive possibility for low-cost field deployment. Furthermore, it might be adapted to smartphones and used as a personal PoC diagnostic (63, 181, 182). Several studies have shown promising RT-LAMP results in SARS-CoV-2, with detection accuracy ranging from 89.9 to 100% (63, 175–177). However, RT-LAMP is challenged by low specificity due to presence of multiple pair primers that may increase non-specific byproduct formation (183). False-negative RT-LAMP results have also been observed for specimens with Ct values above 35 due to low viral RNA; this causes inefficient amplification of the target sequence (175, 183). Sensitivity and specificity of RT-LAMP assays should be evaluated against a range of SARS-CoV-2 viral loads for validation and optimization. LAMP has recently been coupled with nanopore sequencing and CRISPR-based detection platforms (explained below) to boost accuracy and performance (183, 184).

Along with isothermal amplification, another category of nucleic acid tests that could be used for SARS-CoV-2 is the Clustered Regularly Interspaced Short Palindromic Repeats (CRISPR) based method. Use of CRISPR for infectious disease applications has been garnering significant interest over the past few years (185). CRISPR belongs to a family of palindromic nucleic acid repeats found in bacteria, which are recognized and cut by a unique set of effector enzymes known as the CRISPR-associated (Cas) proteins (186). The Cas enzymes are exceptionally sensitive and specific as they can be programmed to identify and cut SARS-CoV-2 RNA sequences (12). Two companies, Sherlock Biosciences and Mammoth Biosciences, are independently exploring these platforms. The Specific High-sensitivity Enzymatic Reporter unlocking (SHERLOCK) assay uses Cas13 (187), and the DNA Endonuclease-Targeted CRISPR Trans Reporter (DETECTR) assay uses Cas12a (188). Cas13a and Cas12a have “collateral cleavage” activity triggered by target-dependent binding between the Cas-guide RNA complex (CRISPR complex) and the target sequence. This event activates the nuclease enzyme activity of the Cas, followed by cleavage of the nucleic acid reporter and generation of a detectable signal (178). Cas13 and Cas12a are activated upon binding to target nucleic acids, RNA and DNA, respectively, where they excise reporter RNA sequences and cut a quenched fluorescent probe to generate a fluorescence signal (187–189). Both tests are low-cost, can be performed in 1 h (188, 190, 191), and have been granted U.S. FDA EUA status (72, 192, 193). The SHERLOCK test demonstrated a sensitivity of 93.1% and a specificity of 98.5% (191), while the DETECTR assay demonstrated 95% positive predictive agreement and 100% negative predictive agreement (188), which makes both strong rapid diagnostic candidates. Another CRISPR/Cas13a system developed by Chinese researchers demonstrated sensitivity approaching a single copy and was highly specific compared to sequencing-based metagenomic and RT-PCR-based assays in a clinical cohort. With reaction TAT of only 40 min after nucleic acid preparation (30 min of DNA amplification by Reverse-transcription Recombinase Polymerase Amplification/RT-RPA and 10 min of Cas reaction), CRISPR is a promising alternative to conventional RT– PCR, particularly in the setting of infrastructure constraints (194). Nonetheless, emerging CRISPR-based methods require careful validation and field testing (195).

Another approach, droplet digital PCR (ddPCR), has been developed to detect SARS-CoV-2 and measure viral load, which facilitates surveillance of inter and intra-case variability (196). ddPCR is based on partitioning the sample into thousands of micro-reactions of defined volume (197). Compared with conventional quantitative PCR, ddPCR has the advantages of being able to perform absolute quantification by using principles of sample partitioning and Poisson statistics. This approach overcomes normalization and calibrator issues associated with qPCR and thus increases precision. ddPCR is also more sensitive for detecting low target copies and relatively insensitive to potential PCR inhibitors (198). Recent studies have reported higher sensitivity and robustness of ddPCR than RT-PCR for detection and quantification of SARS-CoV-2 RNA from purified RNA and crude lysate samples in UTM (196, 199). Digital droplet assays which enable detection and quantification with limited sample processing could potentially be used for monitoring clinical course and convalescence (199).

Genomic sequencing does not play a part in routine SARS-CoV-2 laboratory diagnosis; however, this technique is essential for phyloepidemiological evaluation of changes in the viral genome over time and to trace transmission patterns (67). Sequencing protocols based on Sanger and next-generation sequencing (NGS) (e.g., Illumina and MinION/Nanopore) are being applied to rapidly generate genome sequences (200–202), with the promise that data will inform diagnostic development, epidemiologic investigations, host-virus interactions, viral evolution, pathogenesis, and prevention and treatment targets (67). NGS can also be used to evaluate the host microbiome and co-infection with certain pathogens, which may influence how SARS-CoV-2 infection manifests and results in secondary infections (200). Studies using NGS are sparse in part due to the high cost and the tendency to employ NGS for research purposes as opposed to clinical management (12). In light of ongoing evolution of the SARS-CoV-2 genome, sequencing applications are essential for identifying mutations that may be associated with increasing transmissibility and/or virulence, evading detection by current diagnostics, and escaping antiviral treatment or immunity (203). As of March 2021, several Variants of Concern have been identified as more transmissible (e.g., Variant B.1.1.7 from the U.K.), increasingly resistant to neutralization by monoclonal antibodies, and less susceptible to vaccine induced immunity (e.g., Variant B.1.351 from South Africa, P.1 lineage from Brazil, and Variant B.1.526 from New York City containing the Spike-E484K mutation) (204–207). Given the SARS-CoV-2 genome's evolving nature, genomic surveillance should be conducted at levels that allow early temporospatial identification of new variants.

As of March 23, 2021, the U.K. (*N* = 307,233; 36.50%) and the U.S. (*N* = 200,425; 23.81%) accounted for the majority of all published genomic sequences (N = 841,700) in the GISAID database (7, 135). However, the proportion from reported COVID-19 cases of those two countries (the U.K. = 307,233/4,301,925 = 7.1%; and the U.S. = 200,425/30,576,962 = 0.7%) still lag behind Iceland (4,172/6,119 = 68.2%), Australia (17,674/29,211 = 60.5%), New Zealand (1,211/2,462 = 49.2%), Denmark (50,545/226,777 = 22.3%), and Taiwan (173/1,007 = 17.2%)—the five countries with the highest current proportion of reported sequences (7, 135, 208, 209). Hong-Kong has a sequence reporting rate of 11.0% (1,254/11,398) and documented the world's first confirmed COVID-19 reinfection using whole-genome analysis (7, 89, 135). Genomic surveillance by the South Korea Disease Control and Prevention Agency from January 2020 to January 2021 showed that amongst 2,488 COVID-19 cases, including 648 from abroad, Variant B.1.1.7 and B.1.351 were only identified from international travelers. This supports the efficacy of South Korea's rapid implementation of non-pharmaceutical public health interventions, such as quarantining incoming travelers, for preventing dissemination of SARS-CoV-2 variants (210, 211). Further strengthening of global sequencing capacity will facilitate ending the current pandemic and early detection and management of future outbreaks (208, 212).

**Figure 1:**
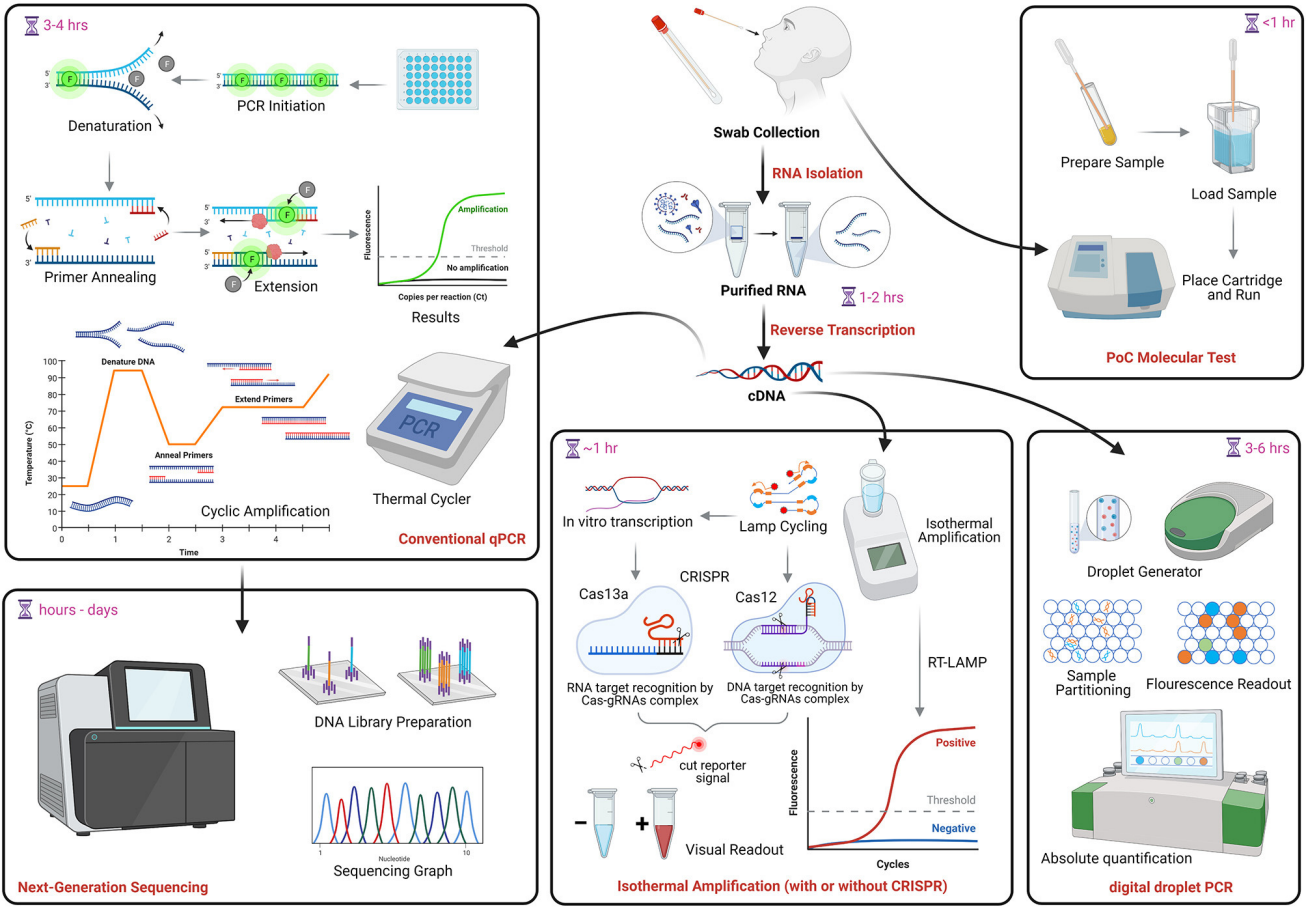
COVID-19 molecular testing. NAAT begins with RNA extraction followed by reverse transcription into complementary DNA (cDNA). The same cDNA can be used for conventional qPCR, RT-LAMP, which can also be coupled with CRISPR technology, and droplet digital PCR. PoC assays (uppermost right) use direct specimen and cartridge-based tests to produce rapid results. The PCR amplification product may be used to generate viral genome sequences (lowermost left). NAAT, nucleic acid amplification tests; qPCR, quantitative polymerase chain reaction; PoC, point of care; LAMP, Loop-mediated isothermal amplification; CRISPR, clustered regularly interspaced short palindromic repeats. Image created in Biorender.com.

Over 300 tests for SARS-CoV-2 NAAT/molecular testing are currently described in FIND (Foundation for Innovative New Diagnostics), a diagnostics resource center established in collaboration with WHO to accelerate development and access to diagnostics as part of the global response to COVID-19. This foundation verifies test LODs using cultured viral stocks from clinical isolates, quantifies using an E-gene standard, and evaluates clinical performance using samples from individuals suspected to have COVID-19 that were tested by in-house PCR. Results are available online at: https://www.finddx.org/covid-19/sarscov2-eval-molecular/. Many molecular and serological PoC tests have also been granted EUAs from the U.S. FDA. Information on these assays can be found at: https://www.fda.gov/medical-devices/coronavirus-disease-2019-covid-19-emergency-use-authorizations-medical-devices/vitro-diagnostics-euas. Figure 1 shows a conceptual overview of COVID-19 molecular testing approaches. Consideration of the pros and cons of each method should guide clinical applications (213).

## *In*-*vitro* Diagnostics: Antibody Assays

Serologic measurement of specific antibodies can be used to assess prior exposure to SARS-CoV-2 and infer potential immunity to the virus. As a diagnostic tool, antibody serology is particularly useful for patients with delayed clinical presentation, typically at least 2 weeks after illness onset (214), who may be missed by NAAT. A report from Singapore demonstrated the utility of antibody measurement in assessing an initially PCR-negative individual who linked two infection clusters (215). Serological data is particularly useful for epidemiologic purposes, such as estimation of the attack rate, R_0_, and case fatality rate (216), and to evaluate the impact of control measures (lockdowns, broad testing, and other policies). Antibody evaluation can also facilitate identification of plasma donors and assessment of vaccine immunogenicity, especially in elderly or otherwise immunocompromised people (214, 216, 217). Cross-reactivity between antibodies to SARS-CoV-2 and other endemic human coronaviruses (CoVs) may enable design of pan-coronavirus therapeutics or vaccines (218, 219). Serological surveillance may also identify potential zoonotic disease transmission from wild-life reservoirs, such as bat-borne coronavirus and influenza virus (e.g., G4 genotype H1N1) (220, 221). However, in a pandemic context where early diagnosis is essential for patient management and outbreak control (222), antibody assays are suboptimal due to delayed seroconversion and performance variability, therefore are not the preferred frontline test (223).

### Antibody Assay Platforms

Currently marketed platforms for serologic evaluation of antibodies include lateral flow immunoassays (LFIA), enzyme-linked immunosorbent assays (ELISA), and chemiluminescent immunoassays (CLIA). These assays rely on similar principles but differ in the method of antibody-antigen binding detection (224). LFIAs, which are small, portable, and suitable for qualitative PoC assessment, result in the appearance of a colored line following the addition of specimen to the strip (225). ELISAs may be qualitative or quantitative and may involve several manual steps, increasing their time to results. Well-plates pre-coated with SARS-CoV-2 spike or nucleocapsid protein are incubated with patient sera, and if antibodies are present, an antibody-antigen complex forms resulting in a downstream fluorescent-based readout (226, 227). CLIAs, also known as chemiluminescent microparticle immunoassays (228), are automated assays that rely on the mixing of patient samples with magnetic, protein-coated microparticles and generate a light-based, luminescent readout (229, 230).

SARS-CoV-2 proteome-based microarrays have more recently been developed for the automated detection of antibodies (231). In contrast to the conventional techniques described above, which test a single target antibody in a single reaction, protein microarrays employ proteome-wide characterization of antibodies in a high-throughput format to generate a more systematic description of antibody binding and viral antigens (232). A similar platform is VirScan, a programmable phage-display immunoprecipitation and sequencing technology platform that was developed in 2015 to explore antibody responses across the human virome. VirScan has been adapted for use with SARS-CoV-2 by employment of a coronavirus oligonucleotide library of 56-mer peptides tiling every 28 amino acids across the proteomes of 10 coronavirus strains, and 20-mer peptides tiling every 5 amino acids across the SARS-CoV-2 proteome. VirScan requires one drop of blood and scans over 1,000 virus strains. A machine learning model trained on VirScan data predicted SARS-CoV-2 exposure with 99% sensitivity and 98% specificity. This type of approach could be very useful for understanding past exposure epidemiology, though it is not yet widely available or suitable for acute diagnosis (233). Biosensors that use polyaniline nanofibers-coated optical fibers for serological measurements are also in development and could eventually be used in a plug-and-play format (234). A microfluidic ELISA system has also been proposed for detection of COVID-19 antibodies via a lab-on-chip platform. Plasma is separated using a microfluidic device and subsequently, antibodies are detected in the separated plasma using a semi-automated on-chip ELISA. Although the automated system is simpler to use than manual ELISA, performance of this platform still needs to be evaluated (235).

In general, LFIAs have lower sensitivities but comparable specificities to ELISAs and CLIAs. In a recent meta-analysis of 40 studies, the pooled sensitivity of IgG or IgM ELISA was 84.3% (95% confidence interval 75.6 to 90.9%), LFIA was 66.0% (49.3 to 79.3%), and CLIA was 97.8% (46.2 to 100%). Pooled specificities ranged from 96.6 to 99.7% (223), consistent with a previous report (224). The low sensitivity of LFIA in this analysis may be related to the use of whole blood, and the use of serum for LFIA and ELISA is likely to increase sensitivity (223). There is high variability in performance amongst commercially available LFIAs (236). This may be related to differences in validation protocols (237, 238), with some studies using archived pre-COVID emergence samples (239–241) and others using PCR negative samples as negative controls (241–243). Validation of immunologic assay techniques following a universal protocol would be very helpful in determining the comparative performance of the assays.

### Spike and Nucleocapsid Protein-Based Antibody Assays

The SARS-CoV-2 spike and nucleocapsid proteins are the primary viral antigens used in currently available antibody assays (244, 245). The spike protein (S) is located on the surface of the virus, where its receptor-binding domain (RBD) attaches to the host ACE2 receptor to facilitate viral entry (246). S is highly immunogenic, and the neutralizing activity of anti-S antibodies has made them the focus of therapeutic and prevention strategies (247). The nucleocapsid protein (N) plays a crucial role in viral replication and assembly (248). N is abundantly expressed during infection, is highly immunogenic, and induces antibody production earlier than S (249). The N gene is reportedly more conserved and stable than S, with 90% amino acid homology and fewer mutations over time, making it a strong candidate for inclusion in vaccines against SARS–CoV-2 (250). However, studies of S, N, and associated antibodies show different results in terms of the superiority of N (251) over S (226). One study has suggested that an S-based assay is more cross-reactive with endemic human coronavirus antibodies than an N-based assay (248). Further studies are needed to characterize antibody dynamics and determine which antigen(s) should be used for monitoring and surveillance purposes.

A major limitation of currently available S-based assays is that they measure total binding antibodies (BAbs) (252) as opposed to neutralizing antibodies (NAbs) alone. Since not all BAbs block infection, these assays do not actually reflect antibody inhibition of SARS-CoV-2 infection, even though some studies have shown that anti-RBD IgG titers correlate with NAbs titers (253, 254). Ideally, assays should specifically assess NAbs as an indicator of protective immunity to facilitate serodiagnosis, evaluation of convalescent plasma therapy, and vaccine development (255). NAbs are conventionally measured by the plaque reduction neutralization test (PRNT) (97), which requires handling infectious SARS-CoV-2 in a specialized BSL-3 containment facility, is labor-intensive, and requires 2–4 days to complete. These limitations make PRNT impractical for large scale applications (252). The pseudovirus-based Virus Neutralization Test (pVNT) utilizes a genetically-modified pseudovirus that mimics SARS-CoV-2 yet is safe to handle and can be evaluated in a BSL2 laboratory (256). Since the broad application of pVNTs is limited by the need for virus and cell culture facilities, the surrogate VNT (sVNT) has been developed to detect NAbs without the need for live virus or cells. sVNTs use purified RBD from the S protein and purified ACE2 to mimic the virus-host interaction in an ELISA plate well. sVNTs can be performed in 1–2 h under BSL-2 conditions and demonstrate 99.93% specificity and 95–100% sensitivity compared with conventional PRNTs (252). Unfortunately, comparative studies of sVNT and PRNT have not clearly defined the sVNT cut-off value in relation to the conventional PRNT titer, though excellent concordance was observed in a small study (257). Further validation between the two assays and using other virus clades is needed to ensure sVNT robustness.

Several point mutations (e.g., Spike-E484K and Spike-S477N) have demonstrated ability to escape neutralization by convalescent sera and monoclonal antibodies (258). Thus, the impact of mutations on SARS-CoV-2 antibody assays should be monitored. Mutations may alter an assay's ability to detect key antibodies, including those to viral spike protein or nucleocapsid. Ongoing evaluation is in progress (259).

### Kinetics of SARS-CoV-2 Isotype Antibodies

Accurate interpretation of serologic testing depends on both antigen specificity and the antibody isotype detected (138). Of the five isotypes, IgM, IgG, and IgA are the primary testing targets (260). IgM is generally produced first because it is expressed on the surface of Naïve B cells prior to isotype switching (261), though IgG conversion prior to and simultaneous with IgM has been seen with COVID-19 (97). The antigen-binding sites of IgM pentamers is not highly specific (262, 263), with one study demonstrating occurrence of SARS-CoV-2 IgM ELISA false-positivity due to mid-to-high levels of rheumatoid factor IgM (22/36 false-positive results). A urea dissociation test was shown to reduce the false-positive rate (264). Low-level cross-reactivity of both IgM and IgG against N and S2-containing antigens from other betacoronaviruses (e.g., SARS, MERS, HKU1, OC43) has been demonstrated in SARS-CoV-2 convalescent blood specimens, although discrimination between COVID-19 cases and negative control is much greater for IgG antibodies than for IgM antibodies (265). In general, IgG is more specific and may appear later in infection (266). IgG is a high-affinity monomer that can directly neutralize microbes as part of the humoral immune response and can be transferred transplacentally from mother to fetus (267, 268). Mucosal IgA responses also play a critical role in blocking viral invasion and replication at mucosal surfaces where SARS-CoV-2 may enter (269, 270). Human breast milk from women exposed to SARS-CoV-2 antigens may contain IgA that protects the infant from infection (271, 272). The role of serum IgA is less clear, but reports suggest it is involved in formation of immune complexes that amplify inflammatory responses (273, 274). Sterlin et al. showed early SARS-CoV-2–specific humoral responses were dominated by IgA antibodies. These were more potent than IgG in neutralizing SARS-CoV-2, highlighting the potential role of IgA during early SARS-CoV-2 infection (275).

Variable kinetics of COVID-19 antibodies have been demonstrated. SARS-CoV-2 IgM may appear and peak earlier than (276, 277), simultaneously with, or after IgG (97, 278). IgA has been detected earlier than IgM or IgG but was found to be cross-reactive with other coronaviruses (279, 280). In a Cochrane Database systematic review of 54 cohorts with 15,976 samples, pooled results for all isotypes showed low sensitivity during the first week after onset of symptoms, rose in the second week, and peaked in the third week. Data on sensitivity of tests beyond 35 days post-symptom onset are inconclusive (281). Serologic antibody testing is useful as a complement to RNA testing, particularly in the later stages as PCR positivity decreases by 2 weeks after symptom onset (282). Antibody kinetics in the setting of COVID-19 re-infection merit further exploration (283).

### Antibody Responses and Disease Severity

Variability in kinetics of SARS-CoV-2 isotype antibodies may be associated with illness severity, age, and comorbidities (276, 284, 285). One study found that IgM and IgG antibodies showed similar kinetics in both non-ICU and ICU patients, with the authors concluding that early class switching of IgM to IgG might predict better outcomes (285). Most studies of antibody responses have occurred in hospitalized COVID-19 patients with moderate to severe illness. Studies in asymptomatic and mildly ill patients have been limited (281), though one study of asymptomatic patients showed SARS-CoV-2 IgG levels (median S/CO, 3.4; IQR, 1.6–10.7) to be significantly lower than in the symptomatic group (median S/CO, 20.5; IQR, 5.8–38.2), with 40% of asymptomatic patients becoming seronegative during early convalescence. One interpretation of these data is that asymptomatic individuals had a weaker adaptive humoral immune response to SARS-CoV-2 infection (278). Other studies have reported later appearance and lower titers of IgA, IgG, and IgM in mild or moderate cases compared to severe cases (260, 277, 281).

Durability of antibodies and how they correlate with immunity are currently unclear (286). A longitudinal population-based study of over 9,000 community residents in Wuhan, China showed that IgG and neutralizing antibodies were relatively stable for at least 9 months, regardless of symptom presence (287). A Danish study observed ~80% protection from re-infection during a second surge (~6 months after initial infection) amongst people with PCR positivity, compared to those who were PCR negative. Protection associated with prior infection decreased to 47% amongst people 65 years or older, supporting prioritization of vaccination for seniors (288). Long term, adequately powered COVID-19 cohort studies are needed to better characterize antibody kinetics as well as correlates of immunity.

## *In*-*vitro* Diagnostics: Antigen Testing

SARS-CoV-2 antigen testing is another type of serologic assay that is attractive as a potential PoC diagnostic. Antigen-based diagnostics detect protein fragments on or within the virus, rather than viral nucleic acids, in specimens collected from NP swabs or nasal cavity (178). This type of testing can detect active infections within 15 min compared to hours with RT-PCR. Therefore, a highly sensitive method that directly detects viral antigens in clinical samples would be a great asset in in the containment of transmission during early infection (289). Viral proteins should be detected by antigen-capture methods (e.g., antibodies, aptamers) which are routinely used for other viral assays, such as human immunodeficiency virus (HIV) and hepatitis B virus (290). Based on previous experience with antigen testing in SARS and MERS, the N protein is considered an excellent target for a diagnostic sandwich assay using monoclonal antibodies. N protein is secreted abundantly during replication and has low cross-reactivity with other human CoVs, such as OC43 and 229E (227, 291). Interestingly, one study that measured serum N protein levels using ELISA in SARS-COV-2 infected patients showed a positivity rate of 76% before antibody was detected, implying that the detection of N protein in serum might be useful for early diagnosis. Although the results are encouraging, this was a very small study (292). Further studies are needed to confirm the results and determine whether infected patients have a higher incidence of viremia in the early stages or whether over-expressed N protein from the lung virus is spilling into the blood.

The widely available SARS-CoV-2 antigen kits use two main approaches: (1) the immunochromatographic (ICT) assay based on colloid gold conjugated antibodies that result in visible colored bands to reflect positivity and (2) the fluorescence immunochromatographic assay (FIA) that provides results via an automated immunofluorescence reader (290). Another approach developed to detect SARS-CoV-2 specific antigen uses nanotechnology in biosensor devices. A field-effect transistor/FET-based biosensing device and fiber-optic absorbance biosensor/P-FAB platform have been developed to detect S and N protein from SARS-CoV-2, respectively (293, 294). Preliminary evaluation suggests these devices are highly sensitive and require no or minimal sample pre-processing (293); however, additional external validation is needed before they can be incorporated into clinical practice.

Several publications on the validation of the antigen kit against the gold standard (PCR) using swab samples showed excellent specificity (99.5–100%) and varying overall sensitivity (11.7–68.8%), with higher viral loads associated with better sensitivity (289, 295–298). This is analogous to the performance of the influenza antigen test in the H1N1 pandemic, where specificity was excellent but sensitivity was low (46.7–53.3%). Suboptimal sensitivity is not unexpected, as low viral loads, consistent with low number of viable viruses and likely low infectiousness, would predispose to false negatives (299, 300). Possible antigen destruction on frozen or repository swab samples may also decrease accuracy (300). According to the manufacturer's instruction for use, nasopharyngeal samples must be fresh and should be tested as soon as possible after collection. Antigen test evaluations performed on leftover sample material after a delay of 1 h to 2 days and storage at 4°C were conducted alongside qRT-PCR (289, 296). These prolonged storage conditions, along with the dilution of samples in transport media, may have impacted assay sensitivity (298). Alternatives to nasopharyngeal swabs, such as sputum or saliva, could also contribute to the variability of results (297, 301).

Although more evidence is needed, data suggest Ag-RDTs are likely to perform well (91–100% sensitivity) in patients with high viral loads (Ct values ≤25 or >10^6^ genomic virus copies/mL) (302), which usually appear in the pre-symptomatic (1–3 days before symptom onset) and early symptomatic phases of the illness (within the first 5–7 days of illness) (303–305). A recent study on community-dwelling subjects with mild respiratory symptoms showed the Ag Rapid Test had 100% specificity and sensitivity above 95% for nasopharyngeal samples when using Ct-values <32 cycles as the cut-off for RT-qPCR test positivity (306). In its September 11th, 2020, interim guidance, WHO recommends use of SARS-CoV-2 Ag-RDTs that meet the minimum performance requirements of ≥80% sensitivity and ≥97% specificity compared to a NAAT reference assay. Testing should be conducted by trained staff in strict accordance with the manufacturer's instructions and within the first 5–7 days following onset of symptoms (302). Patients who present more than 5–7 days after symptom onset are more likely to have lower viral loads and false-negative results with Ag-RDTs (302).

When performance is acceptable, rapid antigen tests can reduce transmission through early detection of highly infectious cases, enabling implementation of targeted isolation and tracking of infectious cases and contacts (307). The excellent specificity of these tests could support public health decisions (298), though the current suboptimal sensitivity suggests that antigen testing may be most useful as an adjunct to the gold-standard RT-PCR (301). According to the updated December 2020 WHO COVID-19 case definition, a person with a positive SARS-CoV-2 Ag-RDT AND who meets either the probable of suspect case definition (high pre-test probability) is classified as confirmed case without RT-PCR confirmation (308). This new case definition is particularly useful in countries with limited molecular NAAT testing. However, because Ag-RDTs can perform differently in manufacturers' trials than in the real world, they merit further comparative evaluation with a standardized validation protocol (309). Additionally, impact of evolving mutations on performance of Ag-RDTs should be anticipated, although it is less likely as most tests target the C-terminus of N gene, which is not a mutation-hotspot (259).

## Future Direction

Availability of established diagnostic technologies has enabled researchers to rapidly adapt them to COVID-19 (114). Lessons from the 2002 SARS outbreak have guided development of COVID-19 detection strategies. Only 3 weeks elapsed from visualization of the virus using transmission electron microscopy to elucidation of the SARS-CoV-2 genetic sequence, while SARS-CoV took 5 months to be recognized (114, 310). This reflects the research community's tremendously accelerated response as well as increases in diagnostic capacity between 2002 and 2020, including accessibility of next-generation sequencing for rapid sequence determination (311). Nonetheless, the ever-expanding panoply of tests requires ongoing optimization. Many need further validation to ensure accuracy, speed, ease of use and broad deployability. Additional research on utility of these diagnostics for zoonotic surveillance may help with mitigation of future epidemics (312).

Control of epidemics requires extensive, ongoing surveillance, and rapid sharing of epidemiological data (313). Smartphones, usage of which has increased exponentially, including in sub-Saharan Africa, can be leveraged for this purpose as they possess connectivity, computational power, and hardware to facilitate electronic reporting, epidemiological databasing, and point-of-care testing (114, 314). Combining diagnostics tools with smartphone integration could support better management, curb transmission of infection and reduce mortality (114).

Safety of laboratory workers who conduct COVID-19 testing is also paramount. Concern for laboratory-associated infection is of particular concern in the setting of Personal Protective Equipment (PPE) shortages, improper microbiological techniques, lack of training, and inadequate decontamination protocols or biosafety measure (315), all of which are more likely to occur when systems are overwhelmed. Optimization of mechanisms to protect laboratory workers should occur in parallel with optimization of COVID-19 diagnostics.

## Conclusion

**Figure 2:**
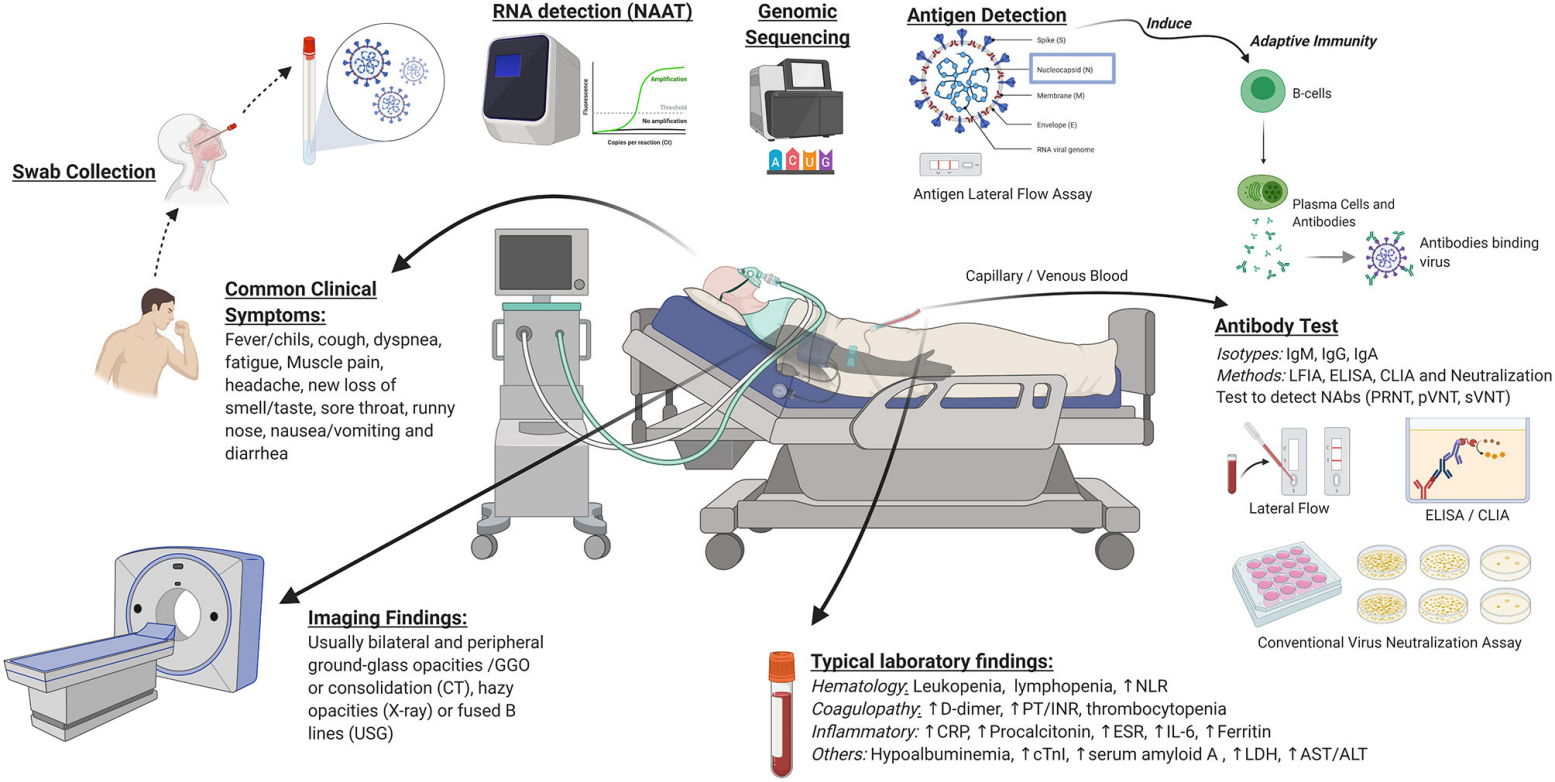
Clinical and *in vitro* diagnostics for COVID-19. Clinical diagnostics consist of common clinical symptoms, imaging findings, and laboratory markers. *In vitro* diagnostics include molecular testing, antibody tests, and viral antigen detection. NAAT, nucleic acid amplification tests; PoC, point of care; CRISPR, clustered regularly interspaced short palindromic repeats; NAbs, neutralizing antibodies; PRNT, plaque reduction neutralization test; pVNT, pseudovirus-based virus neutralization test; sVNT, surrogate virus neutralization test; LFIA, lateral flow immunoassay; ELISA, enzyme-linked immunosorbent assay; CLIA, chemiluminescent immunoassay; NLR, neutrophil-lymphocyte ratio; PT/INR, prothrombin time and international normalized ratio; CRP, C-reactive protein; ESR, erythrocyte sedimentation rate; IL-6, interleukin 6; cTnI, cardiac troponin I; LDH, lactate dehydrogenase; AST, aspartate aminotransferase; ALT, alanine aminotransferase; CT, computed tomography; USG, ultrasound sonography. Image was created in Biorender.com.

Diagnosis of COVID-19 is based upon clinical and *in vitro* approaches. A summary of clinical and *in vitro* diagnostic approaches for COVID-19 is depicted in Figure 2. Basic principles of *in vitro* diagnostics and potential areas for development are listed in Table 1. Selection of the most appropriate diagnostic method depends upon the situation, including patient presentation, timing relative to disease course, laboratory infrastructure, available management options, public health needs, and research agendas. Clinical diagnostic evaluation and antibody and antigen-based assays can complement RT-PCR, the preferred confirmatory diagnostic for COVID-19. While antibody assays are mainly indicated for epidemiologic purposes due to delayed seroconversion, the antigen-based assay may be indicated for rapid identification of highly infectious cases in disease course, which could reduce further transmission. Availability of diagnostic assays is rapidly expanding, as demonstrated by the ever-increasing list of assays granted EUA status by the U.S. FDA. Well-designed validation studies should be conducted to identify products with the best performance and to obtain the data necessary to support licensure. As early diagnosis is essential for patient management and outbreak control, development of rapid, scalable, and high-accuracy PoC assays should be prioritized. Highest priority should be assigned to cost-effective multiplexed PoC tests that identify multiple pathogens.

**Table 1:** *In vitro* diagnostics for COVID-19 and potential areas for development.

***In vitro* diagnostic**	**Currently available assays**	**Brief description**	**Development areas**
Molecular testing, NAAT	RT-PCR assays (conventional or automated). Alternative terminologies include rRT-PCR or RT-qPCR.	• NAAT detects the presence of viral RNA (62) • Purified RNA from clinical specimens is reverse transcribed into complementary DNA (cDNA), then added to a master mix containing target primers and a fluorophore-quencher probe. The RT-PCR process is carried out in a thermal cycler. The fluorophore-quencher probe is cleaved, generating a fluorescent signal that corresponds to the amplified product (63, 114) • While conventional NAAT begins from manual RNA preparation, followed by rRT-PCR; automated systems integrate RNA extraction, purification, amplification, and detection, resulting in rapid, high-throughput results and less contamination (70–72, 74)	• Pre-heating specimens to skip RNA extraction (66–69) • Accuracy with alternative, less invasive specimens (e.g., Saliva) in comparison with standard NP specimens (87–89, 91) • Lower respiratory specimens may provide benefit later in the disease course (94), while non-respiratory specimens may correlate with local symptoms (e.g., stool) or clinical severity (e.g., blood) (99, 103, 133) • Swab pooling to increase testing capacity (93) • Different PCR target regions may affect sensitivity (116, 122–124) • Monitoring effect of SARS-CoV-2 genome mutations on RT-PCR performance (118, 136) • One-step (consolidated RT and PCR) vs. two-step (separate RT and PCR) assays, and uniplex vs. multiplex RT-PCR (63, 65, 114) • Subgenomic RNA and/or Ct value as the surrogate for infectious/live virus (139)
	PoC–Xpert® Xpress SARS-CoV-2	It targets the E and N2 SARS-CoV-2 genes, performed on an automated GeneXpert instrument. LOD 8.26 copies/mL and TAT is 45 min (146)	Further development of Xpert® to detect important SARS-CoV-2 mutations may be needed, as is done for TB (148)
	PoC–CovidNudge	It is based on a fully-automated multiplex RT-PCR targeting seven SARS-CoV-2 gene targets (RdRp1, RdRp2, E-gene, N-gene, N1, N2, and N3). LOD 250 copies/mL and TAT is 90 min (155, 156)	• CovidNudge has low throughput compared with RT-PCR (1 sample per run), multiple instruments may be needed depending on the clinical setting (157) • Studies have only assessed performance with NP/OP swabs (156). Further validation is warranted, and other sample types should be examined
	PoC–TrueNat	This chip-based portable PoC targets SARS-CoV-2 E and RdRP genes. LOD 486 copies/mL and TAT is <1 h (160, 161)	Despite affordability and portability, this technology is low throughput and further external validation studies are warranted (63)
	PoC–ID Now COVID-19	It is based on the Nicking Enzyme-Assisted Reaction (NEAR), which targets the SARS-CoV-2 RdRP gene. LOD 125 genome equivalents/mL and TAT is 5–13 min (149, 150)	Suitability of ID Now as a confirmatory test is uncertain due to a study suggesting low PPA, despite using freshly collected specimens as now recommended by the manufacturers (151, 152)
	PoC–BioFire® Respiratory Panel 2.1 (RP2.1)	It was created by adding primers targeting M and S genes of SARS-CoV-2 to the existing multiplexed BioFire® Respiratory Panel 2 (RP2), which can detect multiple pathogens in a single swab. LoD 500 copies/mL and TAT is 45 min (162, 163)	As RP2.1 detects spike genes, a hotspot for mutation, utility of this PoC test for detection of variants should be routinely assessed.
	PoC–cobas® Liat®	It identifies and differentiates SARS-CoV-2 (targeting ORF1a/b and N genes), influenza A and B virus via multiplex RT-PCR. LoD 12 copies/mL and TAT is 20 min (164)	Since it simultaneously tests for influenza and SARS-CoV-2, thus allowing differentiation between both viruses that may co-circulate in the annual flu season (165). Validation with other multiplexed assays is desired
	PoC–GenMark ePlex	It targets the N gene of SARS-CoV-2 and uses electrowetting and GenMark's eSensor technology based on competitive DNA hybridization and electrochemical detection. LoD 750 copies/mL and TAT is <2 h (155, 171)	The multiplex version (ePlex RP2 Panel) should be further validated with another multiplexed assay (e.g., BioFire® RP2.1 and Cobas® Liat) since NAAT methods differ between those assays
	PoC–Diasorin Simplexa^TM^	It targets SARS-CoV-2 ORF1ab and S genes, can run 8 samples per disc; LoD 500 copies/mL and TAT ~90 min (155, 173, 174)	As it detects the spike gene, a mutation hotspot, utility for detection of variants should be routinely assessed
	RT-LAMP	It detects multiple SARS-CoV-2 genes, including ORF1ab, S, E, and/or N gene, using isothermal amplification, thus does not require thermal cycling (175–178). Real-time results are monitored with colorimetric or fluorescent dyes (43, 180)	• False positives may occur due to presence of multiple pair primers (183), while false-negatives may occur with low viral RNA (175, 183); indicates evaluation should be performed across a range of SARS-CoV-2 viral loads • Smartphone integration and combination with nanopore sequencing and CRISPR-based detection platforms may improve performance (183, 184, 313)
	CRISPR	The guide RNA (gRNA) targets SARS-CoV-2 RNA sequences, which can be recognized by CRISPR-associated (Cas) proteins, result in collateral cleavage of the reporter probes and the appearance of a positive band on the paper strip (178, 187–189)	• Advantages in comparison to RT-PCR include rapid TAT and reduced equipment and reagent requirements (194) • Emerging CRISPR-based methods require validation and additional field testing (195)
	ddPCR	In this digital PCR, the sample is fractionated into thousands of droplets, and the PCR amplification of the template molecules occurs in each droplet, thus allowing for absolute quantification of genomic material (197)	ddPCR assays enable nucleic acid measurement and pathogen diagnosis with limited sample processing, therefore may have a role in monitoring viral load during the disease course and convalescence (199)
	NGS	Sequencing is used to determine the order of the bases within the genome. NGS has three general steps: DNA library preparation, clonal amplification of the library, and DNA sequencing by detecting emitted optical or chemical signals (67, 200)	• Cost is currently high • Potential high utility in genomic surveillance to monitor variants with increased transmissibility and/or virulence, ability to evade detection by current diagnostics, and ability to escape antiviral treatment or immunity (203)
Antibody assays	Serology Assay: • ELISA • CLIA • LFIA	• Antibody serology assays detect antibodies against SARS-CoV-2 (15) • ELISA uses plates pre-coated with viral antigens, such as Spike or Nucleocapsid protein (226, 227), and CLIA uses magnetic, protein-coated microparticles to detect antibodies (228). If the serum contains SARS-CoV-2 antibodies, antibody-protein complexes form and are bound with anti-human antibodies tagged with the enzyme to produce a light-based, luminescent readout (229, 230) • LFIA employs a similar method with sandwich ELISA, but the immunological reaction is carried out on the chromatographic paper by capillary action, results in the appearance of a colored line on the strip (225)	• Serological data is most useful for epidemiologic purposes and may facilitate identification of potential convalescent plasma donors and assessment of vaccine immunogenicity (214, 216, 217), although protective titer is not yet well-defined • Poor sensitivity of LFIA compared with ELISA/CLIA may be associated with use of capillary blood for PoC-LFIA test vs. serum/plasma use on ELISA/CLIA (223) • Possible cross-reactivity with other pathogens and/or rheumatoid factor (248, 264) • Unclear whether Spike Protein-based Assay vs. Nucleocapsid Protein-based Assay has better sensitivity (226, 248) • Seroconversion timing between antibody class varies across studies (276, 281) • Dynamic antibody profiling data between severity stages and the duration of antibody response are not well-established (278, 285) • Theoretical possibility that mutations will affect assay performance (259) • Variable accuracy of results amongst different commercially available kits (236)
	Neutralization Assay: • PRNT • pVNT • sVNT	• NAbs are specific for viral epitopes that mediate entry of the virus into a host cell; thus their presences indicate protective immunity (255) • Conventionally, NAbs were measured by PRNT, in which serial dilutions are incubated on a host cell monolayer for several days to determine final dilution titer at which virus plaque formation is inhibited (97) • pVNT has a similar method but uses other viruses pseudotyped with SARS-COV-2 Spike to mimic the infectious virus (256) • sVNT detects NAbs without the need for live viruses or cells. Using purified RBD from the S protein and the host cell receptor ACE2, this test mimics the virus-host interaction in an ELISA plate well (252)	• PRNT is labor-intensive, requires BSL-3 facility, and takes 2–4 days to complete; it is thus impractical for large scale applications (252). Pseudovirus is safer to handle in a BSL-2 laboratory, but still requires culture methodology (256) • Studies did not clearly define sVNT cut-off value in relation to conventional PRNT titer. Validation with different clades or emerging variants is needed to ensure its robustness (252) • Some studies showed positive correlation between the SARS-CoV-2 viral NAbs titer and the S-RBD–specific IgG, with a NAb titer of 1:80 approximately equivalent to a titer of 1:1,280 for S-RBD-specific IgG (253), or NAb titers 1:160 corresponds to anti-RBD titer ≥1:1,350 (254). Studies differ in specific assay used, so titers between studies may not be equivalent. NAb protective titer is not yet well-defined
Antigen assays	ICT and FIA assay	• Antigen-based diagnostics detect protein fragments on or within the virus (178). They mostly target the C-terminus of N gene/protein via a diagnostic sandwich assay using monoclonal Abs (259) • ICT uses colloid gold conjugated antibodies, resulting in visible colored bands, while FIA is usually read by the automated immunofluorescence reader (290)	• As antigen tests perform best in samples with high viral loads and during the first 5–7 days of symptoms (302), they may be useful for early diagnosis and interruption of transmission (307) • Validation studies needed for fresh vs. frozen swab samples (300), viscous vs. non-viscous specimens (298), NP vs. saliva samples (297) • Performance of antigen assay might be impacted by virus mutations (259)

*NAAT, nucleic acid amplification tests; RT-PCR, real-time quantitative reverse transcriptase polymerase chain reaction; dsDNA, double-stranded DNA; NP, nasopharyngeal; PoC, point of care; LOD, limit of detection; TAT, turnaround time; LAMP, Loop-mediated isothermal amplification; CRISPR, clustered regularly interspaced short palindromic repeats; ddPCR, droplet digital PCR; NGS, next-generation sequencing; ELISA, enzyme-linked immunosorbent assays; CLIA, chemiluminescent immunoassays; LFIA, lateral flow immunoassays; Nabs, neutralizing antibodies; PRNT, plaque reduction neutralization test; pVNT, pseudovirus-based virus neutralization test; sVNT, surrogate virus neutralization test; RBD, receptor binding domain; BSL, biosafety level; ICT, immunochromatographic; FIA, fluorescence immunochromatographic assay*.

## Author Contributions

All authors contributed equally with respect to conception and design of the study, literature review and analysis, drafting, critical revision, editing, approval of the final version, and approve this manuscript for publication. NCI and NIAID collaborators contributed to design of the study, collection, analysis, and interpretation of data, and writing of the manuscript.

## Conflict of Interest

The authors declare that the research was conducted in the absence of any commercial or financial relationships that could be construed as a potential conflict of interest.
